# Pentoxifylline Attenuates Cardiac Remodeling Induced by Tobacco Smoke Exposure

**DOI:** 10.5935/abc.20160057

**Published:** 2016-05

**Authors:** Marcos Minicucci, Fernando Oliveira, Priscila Santos, Bertha Polegato, Meliza Roscani, Ana Angelica Fernandes, Beatriz Lustosa, Sergio Paiva, Leonardo Zornoff, Paula Azevedo

**Affiliations:** Faculdade de Medicina de Botucatu, Universidade Estadual Paulista, São Paulo, SP - Brazil

**Keywords:** Tobacco Smoke Pollution, Ventricular Remodeling, Pentoxifylline, Oxidative Stress, Cardiomyopathies

## Abstract

**Background:**

Tobacco smoke exposure is an important risk factor for cardiac remodeling. Under this condition, inflammation, oxidative stress, energy metabolism abnormalities, apoptosis, and hypertrophy are present. Pentoxifylline has anti‑inflammatory, anti-apoptotic, anti-thrombotic and anti-proliferative properties.

**Objective:**

The present study tested the hypothesis that pentoxifylline would attenuate cardiac remodeling induced by smoking.

**Methods:**

Wistar rats were distributed in four groups: Control (C), Pentoxifylline (PX), Tobacco Smoke (TS), and PX-TS. After two months, echocardiography, invasive blood pressure measurement, biochemical, and histological studies were performed. The groups were compared by two-way ANOVA with a significance level of 5%.

**Results:**

TS increased left atrium diameter and area, which was attenuated by PX. In the isolated heart study, TS lowered the positive derivate (+dp/dt), and this was attenuated by PX. The antioxidants enzyme superoxide dismutase and glutathione peroxidase were decreased in the TS group; PX recovered these activities. TS increased lactate dehydrogenase (LDH) and decreased 3-hydroxyacyl Coenzyme A dehydrogenases (OH-DHA) and citrate synthase (CS). PX attenuated LDH, 3-OH-DHA and CS alterations in TS-PX group. TS increased IL-10, ICAM-1, and caspase-3. PX did not influence these variables.

**Conclusion:**

TS induced cardiac remodeling, associated with increased inflammation, oxidative stress, apoptosis, and changed energy metabolism. PX attenuated cardiac remodeling by reducing oxidative stress and improving cardiac bioenergetics, but did not act upon cardiac cytokines and apoptosis.

## Introdution

Tobacco smoke (TS) is one of the most important risk factors for cardiovascular disease and directly damages the myocardial tissue.^1^ The toxic effects of TS on the heart have been referred as smoke cardiomyopathy.^2^

The potential mechanisms involved in smoke cardiomyopathy and other TS-induced damages include inflammation, oxidative stress, energy metabolism abnormalities, apoptosis, gap junctions remodeling, hypertrophy and angiogenesis.^3-7^ Inflammation exerts a key role in this process.^8^,^9^ One possibility is that inflammation activates enzymes, such as NADPH oxidases, that generate reactive oxygen species (ROS).^10,11^ Inflammation contributes to abnormalities in energy metabolism, which further leads to ROS generation and low levels of adenosine triphosphate (ATP).^2^ During the remodeling process, inflammation, oxidative stress and energy metabolism have been recognized as biochemical abnormalities that induce cellular changes, such as apoptosis.^12,13^ The consequences of the remodeling process are changes in heart size, mass and geometry, which lead to cardiac dysfunction.^14^

In general, smokers have elevated levels of inflammatory cytokines.^8,11^ TNF-α, IFN-γ and ICAM-1 have been considered key cytokines involved in cardiac remodeling, leading to endothelial dysfunction, the intracellular death cascade and ROS production.^9,15,16^

Inflammation constitutes a common feature in the pathogenesis of cardiac remodeling, but anti-inflammatory therapies for heart failure have shown controversial data.^9^

The RENEWAL and ATTACH trials analyzed the effect of the anti-TNF-α drugs, etanercept and infliximab, on heart failure. Neither drug exerted positive effects. In small trials, pentoxifylline (PX), another anti-TNF-α agent, provided beneficial effects.^15,17^

PX is a phosphodiesterase inhibitor with immunomodulatory properties, including the down-regulation of TNF-α synthesis and the inhibition of apoptosis, cell proliferation and thrombosis.^16,17^ PX seems to delay cardiac deterioration by unclear mechanisms, but a combination of immunomodulatory and vasodilatory effects is possible.^17^

Smoking prevention and cessation is the most important strategy for reducing TS induced damage, but given the high number of smokers and the high risk of cardiovascular death in this population, the study of potentially beneficial drugs for minimizing heart damage is truly relevant. PX has been considered a valuable drug, especially under conditions in which inflammation, oxidative stress, apoptosis are involved^18,19^

Therefore, the aim of this study was to investigate the role of PX on the cardiac remodeling induced by TS exposure.

## Methods

The experimental protocol was approved by the Ethics Commission on Animal Experimentation (CEEA) of our Institution. The study complies with the Ethic Principles on Animal Experimentation adopted by the Brazilian Board of Animal Experimentation.

Male Wistar rats weighing 200 g to 230 g were divided in four experimental groups: control group (C), composed of animals not exposed to tobacco smoke; tobacco smoke group (TS), composed of animals exposed to tobacco smoke; pentoxifylline group (PX), composed of animals not exposed to tobacco smoke and fed with 100 mg/kg of pentoxifylline added to chow;^20^ TS-PX group composed of animals exposed to tobacco smoke and fed with chow with the addition of 100 mg/kg of pentoxifylline. All of the animals were observed for two months.

During the first week, the smoke was released at a rate of 10 cigarettes, twice a day in the afternoon, with resting intervals of 10 min. The number of cigarettes was increased to a rate of 40 cigarettes/day (20 cigarettes/30 min in the morning and in the afternoon) until the completion of the study.^21,22^

After this period, morphological and functional analyses were performed, and biological samples were collected for biochemical analyses.

### Invasive systolic blood pressure

The invasive blood pressure measurements were taken by cannulating the femoral artery. The rats were anesthetized with ketamine (50 mg/kg) and xylazine (1 mg/kg) intraperitoneally. Musculature from the inguinal region was dissected to allow for visualization of the left femoral artery.

The femoral artery was dissected and isolated, and the distal portion was tied. An indwelling with polyvinyl chloride catheter (diameter 0.5 mm) filled with heparin solution (500 IU/mL) was placed in the femoral artery, and the catheter was connected to a polygraph (Windograf, GOULD, Ohio, USA). The average of 10 consecutive measurements of diastolic (DBP) and systolic blood pressures (SBP), obtained through graphic records of the polygraph, was recorded. The mean blood pressure was calculated using the formula (SBP+2xDBP)/3. After the pressure measurement, the catheter was removed, and the proximal portion of the femoral artery was occluded.^23^

### Echocardiographic study

Prior to euthanasia, all of the animals were weighed and evaluated with a transthoracic echocardiograph exam, as previously described.^2^ The exams were performed using an echocardiograph (SONO CT HDI-5000, Philips Healthcare, Netherlands, Europe), equipped with a 7.5 MHz phased array transducer. All of the measurements were obtained by the same observer, according to the method recommended by the European Association of Echocardiography.^25^

### In vitro left ventricular function

The isovolumetric isolated heart study was performed as previously described.^24^ Briefly, the entire heart was quickly removed from the chest and transferred to the perfusion apparatus (model 830 Hugo Sachs Eletronick - Grunstasse, Germany). The Krebs-Henseleit solution had the following composition (mmol/L): 115 NaCl, 5.4 KCl, 1.25 CaCl, 1.2 MgSO4, 1.15 NaH2SO4, 1.2 Na2SO4, 25 NaHCO3 and 11 glucose. In the isovolumetrically beating ventricle (200 beats/min), a balloon was inserted inside the left ventricle. The balloon volume was increased in 0.02 mL increments over the end-diastolic pressure range 0-30 mm Hg. The pressure and volume within the balloon were recorded following each increment, and the pressures corresponded to the left ventricular end-diastolic pressure and volume.^24^

### Samples

The animals were euthanized with a large dose of pentobarbital, and their hearts and blood were dissected. A portion of the heart was stored at -80oC. Transverse sections of the left ventricle were fixed in 4% buffered formalin and embedded in paraffin. The rest of the heart was frozen in liquid nitrogen and storage at -80º C freezer.

### Energy metabolism and oxidative stress

Left ventricle samples (200 mg) were used to measure the total protein lipid hydroperoxide (LH) and enzymes from oxidative stress: glutathione peroxidase (GSHPx, E.C.1.11.1.9), superoxide dismutase (SOD, E.C.1.15.1.1), catalase (CAT, E.C.1.11.1.6) and the enzymes from energy metabolism: 3-hydroxyacyl coenzyme-A dehydrogenase (OHADH, E.C.1.1.1.35.), lactate dehydrogenase (LDH, E.C.1.1.1.27) and citrate synthase (CS; E.C.4.1.3.7.), as previously described.^24^

### Western blot for the evaluation of Caspase 3 and BCL-2 expression

The protein extraction was performed with RIPA buffer, diluted in Laemmle buffer, separated by electrophoresis using Mini Protean system 3 Electrophoresis Cell (Bio-Rad, Hercules, CA, USA). The proteins were transferred to a nitrocellulose membrane system in the Mini Trans-Blot (Bio-Rad, Hercules, CA, USA). The membrane was incubated with the primary antibody, which was a caspase-3 antibody (Cell SignalingTechnology^®^) and BCL-2 (B cell lymphoma -2) overnight. The membrane was washed and incubated with the secondary antibody anti-IgG HRP (Cell SignalingTechnology^®^), for 2 h under stirring. Immunodetection was performed using the chemiluminescence according to the manufacturer's instructions (SuperSignal West Pico Chemiluminescent Substrate, Thermo Scientific, USA). The nitrocellulose membranes were exposed to X-ray films (X-Omat AR film) (Eastman Kodak Co., USA) in predetermined time periods for each protein studied. The antibody used for normalization was GAPDH (6C5) rabbit IgG (Santa Cruz Biotechnology, Inc., Europe) at a 1:5000 dilution. Quantitative analyses of the blots were performed by Scion Image software (Scion Corporation, Frederick, Maryland, USA), which is free software available at http://www.scioncorp.com/.

### Statistics

The data are represented as the means and the standard deviation. Variables with non-normal distributions were normalized before comparisons. A two-way ANOVA test complemented by the Holm-Sidak test was used to compare the groups. The two factors considered were TS exposure and PX. If an interaction between the factors was present (p < 0.05), the groups were analyzed independently (C X TS; C X PX; TS X TS-PX and PX X TS-PX). If there was no interaction, marginal comparisons were performed inside the factors TS (with or without TS exposure) or PX (with or without PX). The statistical test shows 3 "P" values: one P value for the interaction between TS and PX [ P (TS x PX)]; another P value for the influence of TS [P (TS)]; and the third one for the influence of PX[P (PX]. The data analyses were performed with SigmaStat for Windows v2.03 (SPSS Inc, Chicago, IL). The significance level was considered to be 5%.

## Results

The mean blood pressure (C = 84 ± 3; PX = 86 ± 4; TS = 93 ± 3; TS-PX = 90 ± 3 mmHg) (p = 0.5), heart frequency and body weight were similar among the groups.

In relation to the effects of smoking, the exposure to TS increased left atrium diameter and area and impaired systolic function, by lowering the positive derivate (+dp/dt) (Table 1).

**Table 1:** Echocardiographic and isolated heart study data

	C (8)	PX (11)	TS (10)	TS-PX (11)	p (TS x PX)	P (TS)	p (PX)
LVDD/BW (mm/kg)	19.0 ± 1.68	19.4 ± 1.13	19.96 ± 1.86	19.98 ± 1.57	0.18	0.70	0.67
LVSV/BW (mm/kg)	7.84 ± 0.77	8.60 ± 1.09	9.03 ± 1.59	8.89 ± 1.28	0.07	0.27	0.43
LVRWT	0.34 ± 0.05	0.37 ± 0.06	0.34 ± 0.05	0.35 ± 0.04	0.46	0.38	0.16
LAD/BW (mm/kg)	10.0 ± 0.48	11.3 ± 1.12	11.7 ± 1.27^*^	11.6 ± 0.96^*^	0.14	0.02	0.18
LAA/BW (cm^2^/ kg)	0.53 ± 0.79	0.56 ± 0.13	0.69 ± 0.06^†^	0.58 ± 0.08	0.03	0.00	0.20
LAA/RAA	1.14 ± 0.15	1.20 ± 0.16	1.31 ± 0.17^*^	1.25 ± 0.13^*^	0.20	0.04	0.07
LVMI (g/kg)	1.46 ± 0.20	1.52 ± 0.24	1.54 ± 0.19	1.49 ± 0.25	0.53	0.96	0.53
EF	92.8 ± 1.71	90.9 ± 3.30	90.3 ± 3,78	90.5 ± 4.56	0.37	0.48	0.20
FS%	58.7 ± 3.30	55.6 ± 5.22	54.7 ± 6.15	55.3 ± 6.84	0.33	0.51	0.97
SP ^‡^(mmHg)	164 ± 14.5	153 ± 3.30	141 ± 7.18	156 ± 9.90	0.26	0.39	0.85
+dp/dt ^‡^ (mmHg/s)	3851 ± 367	3500 ± 185	2725 ± 228^†^	3950 ± 320	0.02	0.30	0.20
-dp/dt ^‡^ (mmHg/s)	2082 ± 311	2417 ± 323	1925 ± 109	2200 ± 129	0.91	0.48	0.26

C: control group; PX: pentoxifylline group; TS: tobacco smoke group; TS-PX: tobacco smoke and pentoxifylline group. Pi: p value for interaction between TS and PX. In the absence of interactions, Pts and Ppx should be considered. Pts- p value for animals exposed to TS (TS+ TS-PX) compared with groups not exposed to TS (C + PX). Ppx: p value for the animals that received PX (PX+ TS-PX) compared with the groups that did not receive PX (C + TS).

* groups exposed to TS are different from the groups not exposed to TS.

† group TS is different from TS-PX and C. LVDD: left ventricle diastolic diameter; LVSV: left ventricle systolic diameter; LVRWT: left ventricle relative wall thickness; LAD: left atrium diameter; LAA: left atrium area; RAA: right atrium area; LVMI: left ventricle mass index; FS: fractional shortening; EF: ejection fraction; BW: body weight. The data are expressed as the mean ± standard deviation. Significance level 5%.

‡ Isolated heart study data from five animals in each group. SP maximum cardiac systolic pressure; +dp/dt: maximum positive derivate; -dp/dt: maximum negative derivate.

The inflammatory data evidenced that the groups exposed to TS presented higher levels of IL-10 and ICAM (Table 2).

**Table 2:** Inflammation data

	C (6)	PX (6)	TS (6)	TS-PX (6)	P TS x PX	P (TS)	P (PX)
TNF-α (pg/mL)	3.00 ± 1.91	1.71 ± 0.70	3.71 ± 2.50^*^	5.50 ± 3.90^*^	0.21	0.07	0.90
IL-10 (pg/mL)	15.1 ± 7.91	9.20 ± 7.30	21.9 ± 16.6^*^	28.1 ± 14.7^*^	0.26	0.02	0.90
ICAM (pg/mL)	4.1 ± 0.40	3.70 ± 0.20	4.30 ± 0.30^*^	4.30 ± 0.60^*^	0.29	0.03	0.20

C: control group; PX: pentoxifylline group; TS: tobacco smoke group; TS-PX: tobacco smoke and pentoxifylline group. Pi: p value for interaction between TS and PX. In the case of Pi<0.05. different letters indicate statistical significance. In the absence of interactions. Pts and Ppx should be considered. Pts- p value for animals exposed to TS (TS+ TS-PX) compared with groups not exposed to TS (C + PX). Ppx: p value for animals that received PX (PX+ TS-PX) compared with groups that did not receive PX (C + TS).

* groups exposed to TS are different from groups not exposed to TS. TNF-α- tumor necrosis factor; IL-10- interleukin 10; ICAM: Intercellular Adhesion Molecule 1

**Figure 1:**
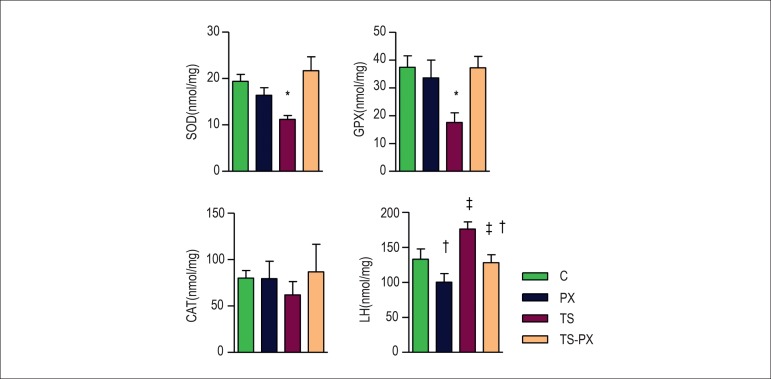
Oxidative Stress. C: control group; PX: pentoxifylline group; TS: tobacco smoke group; TS-PX: tobacco smoke and pentoxifylline group; SOD: superoxide dismutase. CAT: catalase; GSH-PX: glutathione peroxidases; LH: lipid hydroperoxide. There was interaction between TS and PX for SOD (p < 0.001) and GSH-PX (p < 0.001). There was no interaction between TS and PX for LH. LH was higher in the groups exposed to TS (p < 0.001) and lower in the groups that received PX (p < 0.001). * group TS is different from TS-PX and C. ‡ groups exposed to TS are different from groups not exposed to TS. †groups that received PX are different from groups that did not receive PX. The data are expressed as the mean ± 2 SE. Significance level 5%.

Considering oxidative stress, the antioxidants enzyme SOD (C = 20.0 ± 1.93; PX = 16.4 ± 1.65; TS = 11.0 ± 0.89; TS-PX = 21.8 ± 2.96) (p < 0.001) and GSHPx (C = 36.4 ± 4.80; PX = 33.2 ± 6.45; TS = 17.0 ± 3.92; TS-PX = 35.8 ± 5.29) (p < 0.001) were decreased in the TS group (Figure 1). Also, the groups exposed to TS (TS and TS-PX) presented higher levels of LH compared with the groups not exposed to TS (C = 132 ± 14.7; PX = 103 ± 14.5 X TS = 176 ± 11.5; TS-PX = 126 ± 11.9) (Figure 1).

**Figure 2:**
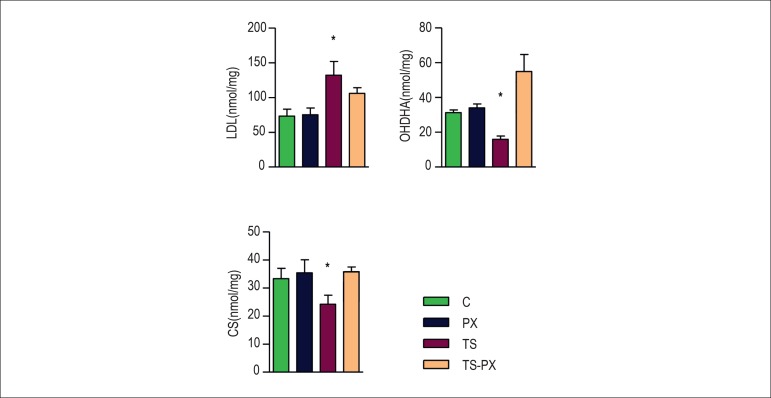
Energy metabolism. C: control group; PX: pentoxifylline group; TS: tobacco smoke group; TS-PX: tobacco smoke and pentoxifylline group. There was interaction between TS and PX for LDH (p = 0.02). OHDHA (p < 0.001) and CS (p = 0.01). LDH: lactate dehydrogenases; OHDHA: 3-hydroxyacyl coenzyme A dehydrogenases; CS: citrate synthase. * Group exposed to TS is different from TS-PX and C. The data are expressed as the mean ± 2SE. Significance level 5%.

Regarding energy metabolism, TS increased LDH (C = 70.7 ± 11.6; PX = 73.5 ± 10.6; 133 ± 21.9; TS-PX = 106 ± 9.88) (p = 0.003) and decreased 3-hydroxyacyl Coenzyme A dehydrogenases (OHDHA) (C = 31.4 ± 1.48; PX = 34.8 ± 2.70; TS = 15.9 ± 2.12; TS-PX = 55 ± 9.88) and CS (C = 31.9 ± 5.00; PX = 35.0 ± 4.75; TS = 24.2 ± 3.57; TS-PX = 36.1 ± 1.71) (p = 0.02) (Figure 2).

**Figure 3:**
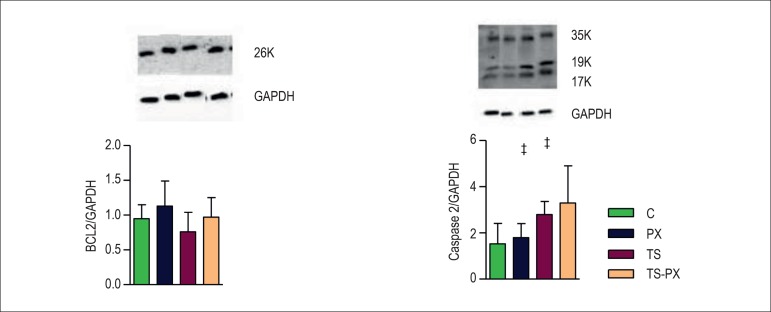
Apoptosis. C: control group; PX: pentoxifylline group; TS: tobacco smoke group; TS-PX: tobacco smoke and pentoxifylline group; GAPDH: glyceraldehyde- 3-phosphate dehydrogenase. BCL-2 (B-cell lymphoma-2). ‡ groups exposed to TS (a) are different from animals not exposed to TS (p = 0.01). The data are expressed as the mean ± 2SE. Significance level 5%.

Finally, TS also increased caspase 3, with none interference in BCL-2. (Figure 3).

About the effects of PX, this drug attenuated the left atrium area and improved systolic function, by increasing +dp/dt, in the TS group (table 1). PX recovered the activities of antioxidant enzymes and improved energy metabolism in groups exposed to TS. Regardless of TS exposition, PX reduced oxidative stress damage, by decreasing of LH (Figures 1 and 2).

PX did not influence inflammation (Table 2) and apoptosis (Figure 3).

## Discussion

The aim of this study was to investigate the role of PX on cardiac remodeling induced by TS exposure. TS induced cardiac remodeling, associated with inflammation, oxidative stress, apoptosis and changed energy metabolism. PX attenuated cardiac remodeling by reducing oxidative stress and improving cardiac bioenergetics.

Considering the consequences of the remodeling process, previous studies have shown that animals exposed to TS presented different remodeling patterns, including hypertrophy, dilation, hypokinesis and dysfunction.^3,21,26^-^29^ The present data showed that TS changed the cardiac morphology, as evidenced by the higher left atrium area and myocytes hypertrophy. In addition, TS lowered the +dp/dt, a parameter of cardiac systolic function under controlled conditions. Therefore, PX attenuated the morphological variables and improved cardiac function, suggesting that this drug has a positive effect on cardiac remodeling induced by TS exposure.

Taking into account the mechanisms of the remodeling process, there is a broad variety of biochemical, cellular, interstitial and molecular abnormalities that contribute to morphological changes and cardiac dysfunction.^30^ Therefore, the rationale for studying the role of PX to attenuate the cardiac remodeling induced by TS is based on the fact that this drug has antiinflammatory, anti-apoptotic, anti-proliferative and vasodilatory properties.^17^ In ischemic heart disease, PX ameliorated inflammatory and apoptotic markers and improved the systolic ejection fraction.^15,16,31^ In addition, preoperative oral PX improved ejection fraction and reduced inflammatory cytokines after coronary artery bypass.^32^ However, the effects of PX on smoke cardiomyopathy are still unknown.

In view of the biochemical parameters, our data showed that TS increased adhesion molecule and Th2 cytokines, such as ICAM and IL-10.^31^ Increased ICAM-1 and IL-10 expression indicate that immunologically mediated injury is present in this model.^16^ In the meantime, PX did not influence these inflammatory parameters. In the current literature, there are some data showing that PX reduced pro-inflammatory cytokines, such as intercellular adhesion molecule-1, and increased anti-inflammatory cytokines, such as IL-10.^15,16^ There are other studies in which PX did not influence the TNF-α pathway.^17^ However, it has been suggested that PX exerts biological and immunomodulatory effects independently of the cytokine levels.^17^

In terms of cellular abnormalities, the present study showed that TS increased caspase-3 activity. PX has been described as an anti-apoptotic and anti-proliferative agent. For instance, PX administration inhibits myocardial apoptosis after adriamycin-induced dilated cardiomyopathy, by blocking the caspase-3 dependent apoptotic pathway.^33^ However, in our study, it did not influence these parameters.

This study showed that PX attenuated oxidative stress and improved energy metabolism. ROS mediated damage was observed in the TS groups, evidenced by low activity of SOD and GPX followed by high levels of LH. PX lowered the LH in the animals exposed to or not exposed to TS. PX increased the SOD and GPX activity in the animals exposed to TS. Oxidative stress is currently thought to have a central role in cardiac toxicity, while antioxidant defenses have a crucial role in protecting the tissue from damage.^11^ It has been reported that PX can significantly attenuate cardiac oxidative stress. Rats fed with western diet presented with mild hypertension and the use of PX further enhanced antioxidant activities and lowered blood pressure.^34^ In addition, the previously described therapeutic effects of pentoxifylline on diabetic heart tissue via NOS, is another important oxidative stress pathway.^35^

Regarding energy metabolism, TS increased LDH, which can be implicated in cardiac damage or in high glucose metabolism. The activities of CS and OHDHA were lowered in the TS group, suggesting impairment of fatty acid oxidation and mitochondrial respiration and density. The abnormal pattern of cardiac bioenergetics showed by these data was also previously observed in models of heart remodeling followed by cardiac dysfunction.^29,36^ Support for the hypothesis that TS impairs cardiac bioenergetics comes from studies where TS induced a significant decrease in respiration and in the phosphorylation rate of mitochondria.^2^ The consequence of this alteration is ROS formation and low energy generation for myocyte relaxation and contraction.

The roles of PX as an antioxidant and a modulator of metabolic energetics have been described in models different from cardiac remodeling. In the context of liver steatosis, the beneficial effects observed in patients receiving PX might be mediated through a decrease in oxidative stress and in the oxidation of lipid products.^37,38^ In a model of paw edema, PX ameliorated the total antioxidant capacity. In the rat brain under toxic stress, PX increased the activity of SOD and GSH-PX, attenuating lipid peroxidation. Considering the mitochondrial function, it is possible that PX improves mitochondrial respiration in the skeletal muscle of atherosclerotic patients.^39^ PX might increase the intracellular levels of cyclic AMP (cAMP) and cyclic GMP (cGMP).^40^

### Study Limitation

The present study has some limitations that should be addressed, due to the lack of evaluation of the entire pathways of apoptosis and inflammation. In addition, different doses of PX were not tested. Finally, it is experimental data that allow us to make hypothesis but not therapeutic recommendation

## Conclusion

In conclusion, the present study revealed that TS induced cardiac remodeling, associated with increased inflammation, oxidative stress, apoptosis and changed energy metabolism. PX attenuated cardiac remodeling by reducing oxidative stress and improving cardiac bioenergetics, but did not act upon cardiac cytokines or apoptosis.
